# Health and social care professionals’ experiences of providing end of life care during the COVID-19 pandemic: A qualitative study

**DOI:** 10.1177/02692163211017808

**Published:** 2021-05-18

**Authors:** Jeffrey R. Hanna, Elizabeth Rapa, Louise J Dalton, Rosemary Hughes, Louise M Quarmby, Tamsin McGlinchey, Warren J Donnellan, Kate M Bennett, Catriona R Mayland, Stephen R Mason

**Affiliations:** 1 Department of Psychiatry, University of Oxford, Oxford, Oxfordshire, UK; 2 Palliative Care Institute Liverpool, University of Liverpool, North West Cancer Research Centre, Liverpool, UK; 3 Specialist Surgery Psychology Team, Oxford Psychological Medicine Centre, Oxford University Hospitals, NHS Foundation Trust, Oxford, UK; 4 Department of Psychology, University of Liverpool, Liverpool, Merseyside, UK; 5 Department of Oncology and Metabolism, University of Sheffield, Sheffield, UK

**Keywords:** End of life, COVID-19, qualitative study, healthcare professionals, social care professionals, experience, supportive care, palliative care, psychosocial wellbeing

## Abstract

**Background:**

Health and social care professionals’ ability to address the needs of patients and their relatives at end of life is likely to have been impacted by the COVID-19 pandemic.

**Aim:**

To explore health and social care professionals’ experiences of providing end of life care during the COVID-19 pandemic to help inform current/future clinical practice and policy.

**Design:**

A qualitative interview study. Data were analysed using thematic analysis.

**Setting/participants:**

Sixteen health and social care professionals working across a range of clinical settings in supporting dying patients during the first wave (March–June 2020) of the COVID-19 pandemic in the United Kingdom.

**Results:**

Participants reported emotional and practical challenges to providing end of life care during the pandemic, including increases in patient numbers, reduced staffing levels and relying on virtual platforms for sensitive, emotive conversations with relatives. Participants were central to promoting connections between patients and their families at end of life and creating opportunities for a final contact before the death. However, the provision of support varied as a consequence of the pressures of the pandemic. Results are discussed under two themes: (1) challenges and facilitators to providing end of life care, and (2) support needs of relatives when a family member was dying during the COVID-19 pandemic.

**Conclusion:**

There is a need for flexible visiting arrangements at end of life during a pandemic. A systems-level approach is necessary to promote the wellbeing of health and social care professionals providing end of life care during and after a pandemic.

**What is already known about the topic?**

- Health and social care professionals are central to addressing the holistic needs of patients and their families as they prepare for end of life.
- Health and social care professionals have encountered challenges in the provision of end of life care during the COVID-19 pandemic.

**What this paper adds?**

- Health and social care professionals highlighted the intensity of workload demands during the COVID-19 pandemic being compounded by increased numbers of dying patients, the deaths of colleagues or their own family members, and reduced staffing levels.
- Physical elements of care were given precedence by health and social care professionals with fewer opportunities for wider, holistic support.
- Tensions were evident between families and professionals, and within healthcare teams, about the appropriate timing of relative visits before the patient’s death.

**Implications for practice, theory or policy**

- Tools such as question prompt lists and charting daily family communication could help promote informative family engagement at times of restricted visiting.
- Clarity in guidance and governance is required to identify when relatives can visit a dying family member in institutional settings during a pandemic, with a clear recommendation that this contact should be facilitated when death is expected in weeks and days rather than hour(s) before death.
- There is a need for visible leadership and support within healthcare teams to promote self-care and reflection, as well as ongoing access to psychological support for health and social care professionals.

## Introduction

COVID-19 is likely to have impacted health and social care professionals’ provision of end of life care. During the pandemic, health and social care professionals have encountered practical and emotional challenges concerning the increase in the number of patients dying,^1^ as well as insufficient resources to provide care.^2^ The pressures of the pandemic may have also affected the wellbeing of health and social care professionals.^3,4^

Health and social care professionals are central to the process of addressing the psycho-social-spiritual needs of patients and their relatives at end of life.^5,6^ During the COVID-19 pandemic, bereaved relatives perceived healthcare teams as instrumental to: ensuring connectedness between patients and their family at end of life through proactive measures such as video and telephone calls; providing relatives with ongoing updates about their dying family member’s declining health; and enabling opportunities for relatives to ‘say goodbye’ when death is imminent.^7,8^ Amidst the challenges of the pandemic, ensuring the needs of families are met at end of life will likely promote better short and long term mental and physical health outcomes for relatives,^9,10^ It is unclear how health and social care professionals navigated the provision of end of life care for families in light of the structural and practical challenges they encountered during the pandemic.^11^ For the purpose of this study, ‘end of life’ refers to the final weeks and days of life.^12^

Exploration of health and social care professionals’ experiences and perceptions offers valuable insights into how professionals’ coped and navigated providing end of life care during the COVID-19 crisis. This will aid our understanding about how health and social care professionals can be best equipped and supported as they provide care at end of life during a pandemic, and will have relevance for clinical practice and policy, now and in the future.

### Aims and objectives

The current study aims to explore health and social care professionals’ experiences and perceptions of providing end of life care during the COVID-19 pandemic in the UK. The objectives of this study are to investigate professionals’:

(1) experiences of providing end of life care and support during the COVID-19 pandemic
(2) perceptions of the needs of relatives when a family member was dying during the COVID-19 pandemic
(3) perceptions of how relatives could be best supported at end of life during the COVID-19 pandemic

## Methods

A descriptive qualitative design using semi-structured interviews. This design is considered most appropriate when exploring individual experiences.^13^ The study is reported in accordance with Consolidated Criteria for Reporting Qualitative Research (COREQ) guidelines.^14^

### Setting

This study was embedded within a national quantitative UK survey of health and social care professionals’ views about end of life care experiences. Participants who expressed an interest in being interviewed after completing the survey were invited to take part in an interview.

### Study population

Individuals were considered eligible if they had a professional role (i.e. doctor, nurse, allied health professional, social worker, chaplain) working with patients at end of life during the first wave of the COVID-19 pandemic (March–June 2020) and resided in the United Kingdom. For clarity and simplicity, the term ‘health and social care professionals’ is used as a collective term to represent the range of professionals involved in supporting families at end of life.

### Sampling

Using convenience and purposive sampling techniques, a range of health and social care professionals working across clinical settings were recruited to the study.

### Recruitment

Eligible participants were contacted by one researcher (RH) via email; 60 potential participants who expressed interest did not respond to the invitation, and two replied stating they were no longer interested in taking part in an interview. Due to the sensitivity of the subject matter, follow-up was not undertaken to ascertain why eligible participants had not responded to the email approach. For those participating, informed written consent was obtained prior to the interview with a study researcher (RH, TM).

### Data collection

Semi-structured interviews were conducted between July and December 2020. A topic guide was developed, informed by the study’s aims and objectives, and the research team, consisting of clinicians and researchers in palliative care, psychology and bereavement. The topic guide was iteratively modified throughout the data collection period to ensure follow-up with categories in subsequent interviews (Table 1). Interviews were completed by two female researchers [RH, TM], who were not known to the participants. Interviews were conducted via telephone (*n* = 11) or Zoom (*n* = 5), audio-recorded and lasted between 32 and 71 min (*mAvg* = 55.1 min). Interviews were completed when no further categories were identified.

**Table 1.** Semi-structured topic guide used to guide the conduct of the study.

Initial topics based on the literature and study aims and objectives • Exploration of professionals’ experiences of providing end of life care during the COVID-19 pandemic.
• Exploration of professionals’ perceptions of how families could be best supported at end of life during the COVID-19 pandemic.
• Exploration of professionals’ perceptions of the psychosocial needs of families when a relative was dying during the pandemic
• Exploration of professionals’ thoughts and feelings about delivering end of life care during the pandemic.
Sample of additional topics as categories were identified
• Professionals’ engagement with psychological services during the COVID-19 pandemic.
• Sharing sensitive and emotional information on the telephone with relatives.
• Tensions within clinical teams about when relatives should spend time with a dying family member at end of life.

### Data analysis

Audio-recordings were transcribed *verbatim* and verified by the research team. The data was analysed using reflexive thematic analysis; a flexible method useful to exploring individual experiences, perspectives and opinions.^15^ Initially, JRH read and reread the transcripts to gain a sense of each professional’s experience. JRH produced written reflections after reading each transcript, outlining thoughts about the individual *story*.^15^ Then, JRH manually coded the data, detailing inductive descriptive codes by marking similar phrases or words from the professionals’ narratives. Reflexive thematic analysis was a useful approach to enabling JRH to reflect and engage with the data, generating themes from the codes using mind mapping techniques. The written reflections aided constructing the themes. Themes were discussed and refined through discussion with all authors.

### Ethical considerations

Health and social care professionals were provided with oral and written information about the study, and provided oral and written consent. Participants were aware of their right to withdraw from the study, as well as the option to pause, reschedule or terminate the interview. Participants were provided with information about support organisations as part of the study’s debrief. Data protection procedures were observed and assurances of confidentially were provided. Ethical approvals were obtained from University of Liverpool Central University Research Ethics Committee [Ref: 7761].

## Results

A range of professionals (*n* = 16) were recruited, including: registered nurses (*n* = 5); team leaders (nurse) (*n* = 1); clinical nurse specialists (*n* = 3); consultant clinicians (*n* = 2); junior doctors (*n* = 2); chaplain (*n* = 1); social worker (*n* = 1); and healthcare assistant (*n* = 1). Sample characteristics are reported in Table 2. The data below represents professionals’ experiences of providing end of life care in hospital (*n* = 10), hospice (*n* = 3) and care home (*n* = 3) settings in the final weeks and days of life. Overall, two themes were identified: (1) challenges and facilitators to providing end of life care during the COVID-19 pandemic, and (2) support needs of relatives when a family member was dying during the COVID-19 pandemic. For simplicity, the terms ‘relatives’ and ‘family’ represent the person closest to the dying patient.

**Table 2.** Characteristics of the 16 health and social care professionals recruited to the study.

Variables	*N*
Hospital based professionals	
Palliative care social worker	1
Palliative care consultant	1
Palliative care clinical nurse specialist	2
Palliative care team leader (nurse)	1
Registered nurse	1
Healthcare chaplain	1
Healthcare assistant	1
Junior doctor	2
Care home based professionals	
Registered nurse	2
Palliative care registered nurse	1
Hospice based professionals	
Palliative care clinical nurse specialist	1
Palliative care consultant	1
Palliative care nurse	1
Location of professional	
England	8
Scotland	5
Wales	2
N. Ireland	1
Gender	
Female	11
Male	5
Time of interview	
July–September 2020	8
October–December 2020	8
Ethnicity of professional	
White (English/Welsh/Scottish/Northern Irish/British)	16

### Theme 1: Challenges and facilitators to providing end of life care during the COVID-19 pandemic

Health and social care professionals highlighted the emotional challenges to providing end of life care during the pandemic, demonstrating a need to adapt throughout the ongoing and changing situation. This is discussed under two sub-themes: (1) emotional demands of providing end of life care during the pandemic, and (2) having end of life conversations with relatives virtually.

#### Sub theme 1: Emotional demands of providing end of life care during the pandemic

Most professionals reported an increase in the number of patient or resident deaths at their place of work, many of whom had tested positive for, or were suspected to have COVID-19. Alongside this, several professionals stated that some of their healthcare colleagues or family members had died after contracting the virus. As a result, professionals described feeling ‘*flat and fatigued*’ with concerns surrounding catching the virus themselves and passing it on to vulnerable family members in their own home.

*“My mother-in-law died at the beginning of the pandemic and it just seems I’m surrounded by death. I know I work in palliative care but at one point we were seeing double the deaths of what we usually would. My father in-law now lives with us and I am petrified that I’ll pass the virus to him.” [Professional 11, palliative care clinical nurse specialist, hospital based]*

Some professionals were redeployed to support patients who were receiving end of life care, which may not have been part of their usual clinical role. Other health and social care professionals had returned to clinical practice from non-clinical roles, retirement or managerial positions to support the health service during the pandemic. While these professionals reported receiving ‘*fast-tracked training*’ to deliver the physical aspects of care at end of life, they felt less equipped and confident to provide psychological care to patients and relatives. Often, redeployed professionals stated they were less comfortable to ‘*open up*’ about how they were coping with the situation with their new colleagues; this was attributed to the difference in their level of connection with unfamiliar peers compared to their established, usual clinical team.

*“I was a bit of an outsider really. I didn’t know the others in this team as well as they knew of each other. I’m used to opening up with my team back in the cancer centre. This was a different team dynamic, and I suppose I just had to get on with it.” [Professional 09, registered nurse, hospital based]*

Health and social care professionals reported feeling supported when they were offered clinical supervision or access to a bereavement counsellor in the healthcare setting to offload the emotional impact of providing end of life care during the COVID-19 pandemic. Supervision was either in person or via virtual platforms. However, it seemed many professionals did not take up these opportunities, perceiving it was ‘*not needed*’ for themselves, but commented that other colleagues might have found it useful.

*“It’s not really my kind of thing (referring to clinical supervision), even though I recognise the importance of it. I just don’t think it’s for me.” [Professional 03, palliative care team leader, hospital based]*

Professionals found it beneficial when they were able to take annual leave for ‘*downtime*’; this gave them the chance to maximise their usual ‘self-care strategies’ such as physical exercise. However, some professionals had few opportunities to take ‘*time off*’ as a result of reduced staff levels due to sickness, redeployment and the increased number of patients receiving end of life care during the pandemic. Consequently, many health and social care professionals reported working longer hours and additional shifts.

*“One Saturday I just felt completely overwhelmed and I just had to say to myself ‘well actually you know you need to have some time off’. But that didn’t happen as there’s only three of us in the team and one was off sick with the virus.” [Professional 01, palliative care social worker, hospital based]*

#### Sub theme 2: Having end of life conversations with relatives virtually

Arising from the public health visiting restrictions implemented in response to the pandemic, professionals reported that relatives were usually informed by telephone that their family member was going to die within weeks or days. Many professionals were concerned about whether a relative would have other people around to provide comfort when they received this call. At times, health and social care professionals working in hospital settings felt it was challenging to share this news with relatives, as they had not had an opportunity to develop a rapport or relationship with them, having never met physically or virtually.

*“I felt I was apologising so much because it felt so inadequate. I knew little about the patient or her family, or what they were going through, and yet here I was about to tell them devasting news.” [Professional 15, palliative care consultant, hospital based]*

Often, health and social care professionals reported it helpful to have a list of prompts as they shared the news on the telephone with relatives that death was imminent. Professionals perceived it was good practice to start the call by introducing themselves and informing the relative that they had been caring for their family member. Professionals felt it was important to provide relatives with an update surrounding their family member’s condition, such as changes to their physical condition and symptom management. It was considered necessary to use clear and unambiguous language with relatives when telling them that their family member was ‘*expected to die soon*’, avoiding vague phrases such as ‘*we have done all we can*’. Communicating the uncertainty around prognosis was a challenge for some health and social care professionals, especially over the telephone. Professionals stated it was useful to explain to relatives that ‘*it may be difficult to predict when the death may happen, but we will keep you updated as much as possible*’. Health and social care professionals perceived relatives had found it helpful when the professional (1) offered to share this news with another family member to facilitate support within family groups, and (2) created opportunities for questions to be asked.

*“Looking back, it was easier to have these conversations face to face as the family are usually there and you can provide a bit more emotional support. We quickly realised once that phone call ends, that’s it for probably a few hours, at least. It was important for us to get it right from the beginning.” [Professional 06, palliative care consultant, hospice based]*

Throughout the end of life period, most professionals were conscious that visiting restrictions meant relatives were less able to observe changes in their dying family member’s health. Consequently, health and social care professionals tried to provide increased telephone calls to keep relatives updated. In addition, there was an absence of end of life volunteers in the care settings to help provide this aspect of supportive care, such as sitting with the dying individual. Some professionals described a ‘communication chart’ being developed and deployed at their place of work to record when and who contacted relative(s) and the depth of information provided. This ‘chart’ was perceived by professionals as a practical audit trail for the healthcare team to ensuring communication with relatives was prioritised. Other professionals felt it was helpful when families nominated one ‘spokesperson’ to contact the healthcare team for updates regarding their dying family member as they were receiving high volume of calls due to reduced visiting.

*"Understandably, relatives had a need for more information on the telephone when they weren’t visiting. Normally there would have been huge numbers of volunteers who would have helped with these calls, but that was gone. It was unmanageable for some teams.” [Professional 13, healthcare chaplain, hospital based]*

### Theme 2: Support needs of relatives when a family member was dying during the COVID-19 pandemic

Most health and social care professionals reported an awareness of relatives’ needs at end of life and the importance of providing supportive care to the whole family in the final weeks and days of life. However, it seemed the provision of this care in clinical practice varied and was often impacted by the pressures facing professions during the pandemic. As a result, many health and social care professionals had to concentrate on providing physical elements of care such as pain and symptom management, with opportunity to engage in family-focused psychosocial support often limited. These issues are discussed under two sub-themes: (1) maintaining a connection with the family member in their final weeks of life, and (2) final contact and ‘saying goodbye’ to the dying family member.

#### Sub theme 1: Maintaining a connection with the family member in their final weeks of life

Health and social care professionals felt it was important for relatives to stay virtually connected with their dying family member in the final weeks of life. In the absence of visiting, some professionals reported facilitating video calls between relatives and the dying family member, which were either suggested to the relative by the professional or requested by the family. Often, it appeared these video calls took place on a professionals’ personal mobile phone as there was a lack of available devices in the hospital or residential home. Virtual interactions were considered vital by some professionals as relatives may have not seen their family member for weeks or months due to the pandemic and ‘*allowed them to see for themselves their loved-one was doing okay*’. Some health and social care professionals felt video calls enabled a sense of connectedness between the patient and their usual family life; one professional reported facilitating a Zoom call to enable a patient to be part of their grandchild’s birthday. Other health and social care professionals reported that videos calls were rarely offered or facilitated because professionals or families feared it could be too distressing for either the patient or relative. This was reported to be a particular concern when the patient and/or relative had dementia or a form of cognitive impairment.

*"There was a man who I was with yesterday and it was his 66* ^th^ *wedding anniversary, so we managed, well actually I had to get my own phone and I was able to FaceTime his son, who was with his wife so they could speak together on their anniversary.” [Professional 14, palliative care clinical nurse specialist, hospital based]*

On rare occasions, some relatives were able to spend time with their dying family member in the final weeks of life. This was dependent on the patient having a single-room, (as there was ‘less risk’ of transmitting COVID-19 to other patients or residents) and being thought not to have COVID. Where this was possible, health and social care professionals reported relatives were able to provide personal aspects of care to their dying family member such as giving them a drink or helping with bathing. One professional described a young child being able to spend time with his father by watching TV together.

*“Because he [patient] was in a side room on his own, his little boy was coming in and spending time with him. We even set up a DVD for them to watch.” [Professional 09, registered nurse, hospital based]*

#### Sub theme 2: Final contact and ‘saying goodbye’ to the dying family member

Consistent with the previous sub-theme, maintaining a connection between the patient and their family was viewed as crucial by professionals as death approached. Health and social care professionals believed it was important for relatives to have final contact with their dying family member; many would not have seen them for weeks or months due to COVID-19 restrictions, and professionals felt that it would be helpful for navigating their bereavement experience. Despite this belief, tensions were evident within healthcare teams about when to invite relatives to spend time with their dying family member. While some professionals interpreted COVID-19 visiting restrictions at end of life to mean when death was expected within hours, others understood this as the final weeks or days of life. Often, health and social care professionals described relatives *‘pleading’* with them for the chance to spend time with their family member before they died. At times, professionals reported they ‘*got it wrong*’ as often death happened sooner than expected, and relatives did not get time to visit their dying family member. This was irrespective of whether the patient/resident had tested positive for, or were displaying symptoms of, COVID-19. More often, health and social care professionals thought it would have been best if relatives had spent time with their dying family member when they were in a better state of health, that is, during the final days of life, rather than hours before the death.

*"I think you’ve got to look at the bigger picture. These people are not getting proper closure. They’re not getting time to say the things they need to say. I felt the risk of visiting was low as she didn’t have the virus, but that decision was out of my hands.” [Professional 05, palliative care nurse, care-home based]*

Health and social care professionals stated that visiting restrictions at end of life meant that usually only one relative could spend time with the dying family member as death became imminent, presenting families with a difficult choice. Some health and social care professionals felt it was inappropriate for families to make this decision, often reflecting how difficult it would be for their own family if they were faced with this situation. On occasions, professionals reported ‘*breaking the rules*’ to allow other relatives (usually two to three) to spend time with their dying family member.

*“I felt that it was really mean. The lady had two daughters and was unfair for them to decide who gets to see their mum before she died. I told them both to come and just blame it on me when they got to the hospital door.” [Professional 04, junior doctor, hospital based]*

Health and social care professionals usually met relatives at the reception area when they came to the clinical or care home setting to spend time with their dying family member. This was considered important so the professional could prepare relatives for ‘*what they were about to see*’, as well as helping the relative put on the appropriate personal protective equipment. Some professionals felt they needed to stay in the room/area with the relative when they were spending time with their family member as there was no one else around to provide emotional support. This was not always possible for professionals due to ongoing clinical demands. Most professionals felt this was a ‘*private moment*’ between the dying family member and their relative; perceiving it would be too intrusive to be present and ‘*left them to it*’.

A number of relatives were unable to spend time with their dying family member at end of life because they were ‘shielding’, or lived too far away. In the absence of visiting, some professionals felt it was important to ask relatives if and how the healthcare team could facilitate the spiritual and emotional needs of the patient and family when death was imminent. On occasion, professionals reported spending *‘more time than usual’* with the patient and holding their hand while they slept, playing the patient’s favourite music through the health and social care professionals’ personal mobile phone or MP3 players that were donated to the caring facility, or reading verses from religious texts.

*“His wife was unable to come in as she was shielding. I asked if she wanted me to do anything, and she wanted me to sit with him and hold his hand when he was imminently dying. The ward was busy, well to be honest it was always busy, but no-one else was around to do it.” [Professional 03, palliative care team leader, hospital-based]*

## Discussion

Multiple challenges were faced by health and social care professionals during the pandemic, including maintaining care standards for dying patients and their relatives, and managing their own emotional well-being. Many professionals reported struggling with the deaths of colleagues and their own family members. Equally, health and social care professionals expressed concerns regarding vulnerability to risk of infection, and the possibility of being responsible for passing COVID-19 on to their loved ones. This study also highlights particular issues for health and social care professionals in navigating support for families, such as relying on virtual platforms for sensitive and emotive end of life conversations with relatives, as well as enabling a connection between the patient and their relatives throughout the end of life period. Other impacts of the pandemic faced by healthcare teams included practical pressures such as increased workloads and reduced staffing within clinical teams. Where resource and time was limited for health and social care professionals, it seemed symptom management often took precedence over psychosocial aspects of care at end of life. These findings suggest that the practical and emotional challenges encountered by professionals during the pandemic impacted on their wellbeing, which in turn may have impacted on their provision of patient care.^16,17^

Health and social care professionals felt it was important for relatives to be physically present with their family member before they died to ‘say goodbye’. This concurs with the reports from relatives bereaved during the COVID-19 pandemic.^7^ It seemed professionals were presented with a moral dilemma surrounding what they believed was best for families and what could be offered under COVID-19 restrictions. It may be that participants had different interpretations of the term ‘end of life’; for some ‘end of life’ is perceived as hour(s) before death, whereas others understand this to be the final weeks or days.^12^ Earlier face-to-face contact (before the final hour(s) of life) may mean that relatives are able to spend time with the patient whilst they are still conscious and able to communicate.^9,18^ This can aid a better end of life experience for families and facilitate coping in their grief.^9^ Due to the unpredictability of when a death may happen,^19^ recognition of the importance of flexible visiting arrangements at end of life in light of pandemic-related restrictions may reduce tension within clinical teams, and avoid health and social care professionals feeling they have to ‘*break the rules*’ in order to deliver optimal family-centred care.

Literature highlights that the provision of psychological support and supervision may promote health and social care professionals’ resilience to providing end of life care,^20^ as well as reducing the potential for burnout.^21–23^ While health and social care professionals described the provision of end of life care during the pandemic as emotionally challenging, most professionals did not access the psychological support available to them. It is possible that health and social care professionals viewed themselves as care ‘givers’ rather than ‘receivers’, which made it difficult for some to acknowledge their own psychological support needs.^24^ During the peaks of the pandemic, staff priorities may have been on ‘the need to keep going’ in order to deliver care to patients. High levels of anxiety over extended periods may have meant there was insufficient time to return to a resting/soothe state.^25^ Engagement with psychological support may be higher during the recovery phase of the pandemic when there is greater capacity for reflection.^25^

Some redeployed professionals reported they were less comfortable to make use of psychological support as they had a lack of rapport with their new team members, highlighting the importance of pre-existing relationships within healthcare teams.^26^ While recognising the operational necessity of health and social care professionals redeployment, it is important not to underestimate the impact of this dislocation from the informal, valued support of their usual clinical team for professionals’ emotional wellbeing and coping. Furthermore, moving to an unfamiliar specialty may have left some health and social care professionals feeling deskilled and vulnerable. These consequences of redeployment could be mitigated in the future by clear induction into the new specialty or team and establishing a ‘buddy system’ between junior and senior professionals.^27^

It seemed ‘time off’ provided respite for health and social care professionals, but was practically challenging due to increased patient numbers, and staff rotas under pressure from staff sickness. Literature highlights that clinicians report better scores on job satisfaction, stress levels, general health and productivity when they have opportunities for ‘downtime’ including annual leave.^28,29^ Strategic leadership is vital in enabling and promoting self-care for all members of staff,^30^ for example through protected time to attend clinical reflection or Schwartz rounds.^31^ The active participation of senior professionals is essential for these activities to become embedded in wider team practice.^31,32^ The exhaustion funnel may be a useful tool in explaining the importance of self-care to clinicians.^33^ Practical applications could include encouraging professionals to be more mindfully engaged when taking a break, rather than mentally planning the next part of their shift or reviewing previous decisions in a shift. These factors may aid posttraumatic growth for health and social care professionals.^34^

### Strengths and limitations of the study

The study findings are reflective of health and social care professionals’ experiences working in various settings (hospital, hospice and care home) during the COVID-19 pandemic. This study does not comment on how psychosocial and holistic care changed during the pandemic for health and social care professionals and their patients in this study due to the differences within NHS trusts and national COVID-19 restrictions. Future work should focus on the psychological phases of the pandemic (preparation/action/recovery) such as investigating if during the action phase immediate clinical needs were prioritised while in the recovery phase there may have been capacity to consider the broader psychosocial aspects of care. Most of the sample are representative of professionals working in palliative care settings; future studies should consider a range of non-specialist palliative health and social care professionals who are involved in the provision of end of life care. Results may have important implications for other professionals irrespective of their clinical role. Findings are limited to clinicians who identified as White British working in the United Kingdom during the COVID-19 pandemic. Due to higher rates of COVID-19 in minority groups, future research should investigate the experience of professionals from these groups.

## Conclusion

Health and social care professionals faced practical and emotional pressures in providing end of life care during the COVID-19 crisis. This included increases in the number of patients receiving end of life care, reduced staffing levels due to sickness and redeployment, as well as the deaths of colleagues or their own family members. While clinicians played a central role in the psychological care of patients and their relatives at end of life, this was adversely affected by the pressures of the pandemic. Clear guidelines are needed to outline when relatives can visit a dying family when they are receiving end of life care in an institutional setting; this will minimise dilemmas and tensions between health and social care professionals, relatives and within healthcare teams. To promote the well-being of clinicians during and after a pandemic, there is a need to foster working environments that advocate self-care and reflection as well as access to psychological support.
